# Effect of Marker Ball Positioning on Templating Accuracy in Hip Arthroplasty: A Closed-Loop Audit

**DOI:** 10.7759/cureus.114297

**Published:** 2026-08-10

**Authors:** Heather Tetley, Marcus Cox, André Fernandes, Shaul Gordon, Jamie C Routledge, Smit Shah, Charlie Jowett

**Affiliations:** 1 Trauma and Orthopaedics, York and Scarborough Teaching Hospitals NHS Foundation Trust, York, GBR; 2 Trauma and Orthopaedics, Huddersfield Royal Infirmary, Huddersfield, GBR; 3 Orthopaedics, The York Hospital, York and Scarborough Teaching Hospitals NHS Foundation Trust, York, GBR

**Keywords:** digital templating, hemiarthroplasty, hip fracture, marker ball, total hip replacement

## Abstract

Introduction

Positioning of external calibration devices (ECDs) in anteroposterior (AP) pelvic radiographs influences magnification correction and therefore the accuracy of digital templating in hip arthroplasty. Orthopaedic departments rely on radiology teams to accurately position ECDs on pre-operative radiographs, but due to multiple factors, placement is not always satisfactory, which may incorrectly guide selection of the hip prosthesis size. This closed-loop audit assessed the accuracy of templating femoral head size in patients with intra-capsular neck of femur (NOF) fractures before and after an instructional intervention for radiology staff on optimal marker ball placement.

Methods

A closed-loop retrospective audit was performed at York District Hospital, UK. Data were collected from September to December 2023 and March to May 2024. Inclusion criteria were trauma patients undergoing hip arthroplasty who had a templated pre-operative pelvic radiograph via TraumaCad software (Brainlab, Munich, Germany). The agreed audit standard was that 100% of pre-operative pelvic radiographs should contain a correctly positioned marker ball. The marker ball is a metal sphere, attached to a stand, used whilst obtaining a pelvic radiograph, as a calibration reference to accurately determine the true size and magnification of anatomical structures on the radiograph; this is used by the TraumaCad software to assist the template. Mean absolute head sizes were compared between digitally templated head sizes and implanted head sizes via intra-operative prosthetic stickers. Following the first cycle, results were presented at the departmental audit meeting, and a teaching session on marker ball positioning was delivered to radiology staff. The second cycle was conducted post intervention, and mean differences between templated and actual head sizes in both cycles were compared using a parametric unpaired t-test, with significance set at p < 0.05.

Results

In the first cycle, 52 patients met the audit inclusion criteria, of whom 50 (96.2%) had pre-operative pelvic radiographs with a fully visible marker ball. These 50 cases were included for accuracy analysis. The mean difference between templated and implanted head size was 2.82 mm (range: −5 to +11 mm); 24 patients (48%) received a larger head size than templated, 18 (36%) a smaller size, and six (12%) received the same size. Post intervention, 43 patients met the inclusion criteria in the second cycle. Of these, 37 (86.0%) had a fully visible marker ball and were included for analysis. The mean difference between templated and implanted head size decreased to 1.51 mm (range: −4 to +7 mm); 16 patients (43%) had larger actual head sizes, 12 (32%) had smaller, and nine (24%) had identical sizes. Statistical comparison demonstrated a significant improvement in templating accuracy between audit cycles (p = 0.008). However, despite the improvement in accuracy of head size selection, compliance with fully visible marker head balls in the radiograph decreased from 96.2% to 86.0%.

Conclusion

This quality improvement project demonstrates that a targeted educational intervention can significantly enhance the accuracy of radiological marker ball placement, leading to improved templating precision in hip arthroplasty.

## Introduction

Digital templating is widely used for pre-operative planning in hip arthroplasty and the management of intra-capsular neck of femur (NOF) fractures. Accurate templating assists in predicting prosthetic head, stem, and cup sizes, facilitates appropriate stock preparation, and reduces intra-operative uncertainty [1]. Calibration error of external calibration devices (ECDs) significantly affects templated component sizing, with poorly positioned devices negatively impacting accuracy [2]. Several types of ECDs exist and are widely used, including the 25-mm spherical marker ball, dual scale calibration markers (DSCM) [3], acetate templates [4] and double-marker systems such as the KingMark [5].

Templating accuracy using a marker ball depends on precise radiographic calibration. Correct placement requires the marker to be positioned parallel to the coronal plane and fully visible on the radiograph, equidistant between the femora and at the level of the greater trochanters (GTs) on a well-centred anteroposterior (AP) pelvic plain radiograph [6,7]. Incorrect positioning introduces substantial magnification error, which may result in inaccurate implant sizing and increased risk of postoperative complications such as implant loosening, fractures, and limb-length discrepancy [8]. Even small positional deviations can lead to measurable differences in estimated component size [9]. Achieving correct placement can be challenging in frail NOF patients, who may be in significant pain or unable to maintain optimal positioning, leading to variability in clinical practice.

Frequent inaccuracies in marker ball placement were identified within our department. This prompted a closed-loop quality improvement audit assessing the accuracy of femoral prosthetic head templating before and after the education of radiology staff regarding optimal marker ball positioning during imaging. The aim was to assess whether more exact positioning of the marker ball would positively impact head size accuracy.

## Materials and methods

A retrospective, non-randomised closed-loop audit was performed at York District Hospital, UK. Data were collected for the first cycle from September to December 2023 and for the second cycle from March to May 2024 following an educational intervention. Inclusion criteria were all hemiarthroplasties or total hip arthroplasties (THAs) for intra-capsular neck of femur fractures where the pre-operative pelvic radiograph had been templated using TraumaCad (Brainlab, Munich, Germany), a computer-aided design software designed to help plan an operation, increasing efficiency and accuracy. This permitted direct comparison between radiologically templated and implanted head sizes. The TraumaCad software was solely utilised by operating surgeons of registrar and consultant level to template radiographs pre-operatively. The audit was registered and approved prior to commencing data collection by the quality governance department at York District Hospital, where all the procedures were performed.

Templated radiographs were reviewed using the York Hospital electronic patient record by two authors working as orthopaedic senior resident doctors (one per cycle due to rotational placements); optimal marker ball positioning was assessed according to the parameters detailed in the introduction, and radiologically templated head sizes were compared with implanted sizes recorded on intra-operative prosthetic stickers. Data were collated in a spreadsheet, and mean differences were calculated for each cycle.

The departmental audit standard required that 100% of pre-operative pelvic radiographs contain a correctly positioned marker ball, defined as fully visible, inferior to the pubic symphysis, and equidistant between the femora at the level of the GTs.

Following presentation of the first-cycle findings at the local trauma and orthopaedics audit meeting, radiology staff received targeted teaching delivered by two authors. This teaching lasted 30 minutes in total and included a session on discussing the current method of placing the marker ball within the department, a workshop-style instruction on correct marker ball placement relative to the GT as set out on the posters (Appendices) with any questions pertaining to this, followed by distribution of the educational posters including displaying them on the walls of key rooms in the radiology department (Appendices). The second cycle was undertaken post intervention, and findings were again presented locally.

Mean differences between templated and implanted head sizes were compared using a parametric unpaired t-test. A two-tailed p-value <0.05 was considered statistically significant.

## Results

In the first audit cycle, 52 patients were identified who had a templated pelvic X-ray and had undergone a hip hemiarthroplasty or THA for trauma in September to December 2023. Of these, only two templated pelvic X-rays did not have a marker ball present (Table 1). Therefore, 50 patients met the inclusion criteria in the first round of having a templated X-ray and a fully visible marker ball; the two patients missing a marker ball were excluded. Templated and implanted head sizes were compared in these 50 patients, and the mean difference in head size was calculated as 2.82 (range: -5 to +11) (Table 2). A total of 24 patients had a larger actual head size (mean: +4), 18 patients had a smaller head size (mean: -2), and six patients had the same head size templated and implanted.

**Table 1:** Audit standard outcomes for the first audit cycle. Data are represented as percentages (%) and the number of patients meeting the inclusion criteria out of the total number of patients. Compliance is shown as the number of patients (N) and the corresponding percentage. Statistical significance is reported at p < 0.05.

Audit standard	Compliance	Target
Marker ball present	96.2% (50/52)	100%
Marker ball present AND fully visible	96.2% (50/52)	100%

**Table 2:** Mean differences in head size pre- and post-intervention.

	Mean difference in head size	Range
Pre-intervention	2.82	-5 to +11
Post-intervention	1.51	-4 to +7

The results of the first cycle were presented at the local audit meeting and to the Radiology Department at York Hospital, alongside teaching on correct marker ball placement. Instructional posters were created and distributed throughout the Radiology Department (Appendices).

Accuracy of head size was then re-audited in the second cycle a few months later. A total of 43 patients were identified who had undergone a hemiarthroplasty or THA for trauma and had a templated pre-operative pelvic radiograph between March and May 2024. Of these, three templated pelvic X-rays did not have a marker ball present, and a further three X-rays had a marker ball, but it was not fully visible (Table 3). Therefore, 37 patients met the inclusion criteria in the second round; six patients with absent or partially visible marker balls were excluded. Templated and implanted head sizes were compared in these 37 patients, and the mean difference in head size was calculated as 1.51 (range: -4 to +7) (Table 2). Sixteen patients had a larger actual head size (mean: +2.25), 12 patients had a smaller head size (mean: -1.67), and nine patients had the same head size templated and implanted.

**Table 3:** Audit standard outcomes for the second audit cycle. Data are represented as percentages (%) and the number of patients meeting the inclusion criteria out of the total number of patients. Compliance is shown as the number of patients (N) and the corresponding percentage. Statistical significance is reported at p < 0.05.

Audit standard	Compliance	Target
Marker ball present	93.0% (40/43)	100%
Marker ball present AND fully visible	86.0% (37/43)	100%

Statistical analysis

In the first cycle, the mean difference between templated and implanted head size was 2.82 mm (range: -5 to +11 mm); in the second cycle, this fell to 1.51 mm (range: -4 to +7 mm). Comparison of the two cycles using an unpaired t-test showed a statistically significant improvement in templating accuracy (t = 2.720, df = 83, p = 0.008). The mean difference between cycles was -1.31 mm (95% CI: -2.27 to -0.35 mm), indicating a smaller average discrepancy between templated and implanted head size in the second cycle.

## Discussion

This closed-loop audit demonstrates the significant improvement in accuracy of THA and hemiarthroplasty templating that can be achieved with simple educational interventions. Following targeted radiology teaching, templating accuracy improved significantly, and it is highly likely that more accurate marker ball placement on initial pelvic X-rays has played a major role in this. Specific marker ball placement at the level of the GTs, with a fully visible ball, will lead to more accurate correction for magnification when estimating prosthetic femoral head size [10]. This significant improvement in templating accuracy will have a positive effect on clinical care, including improved pre-operative checks of prosthesis availability. This is especially useful at extremes of head sizes; more accurate pre-operative planning can reduce the chance of a very large or very small prosthetic head size being unavailable intra-operatively. Accurate templating can also impact leg length discrepancy and femoral offset, as well as reducing post-operative dislocation risk [11-13]. The use of marker balls and other ECDs varies between centres, and multiple publications have suggested that simply adjusting the magnification percentage to a specific amount (e.g., 21%) is an effective alternative that could negate the need for any calibration device [14-17]. However, it is widely acknowledged in the literature that the use of ECDs impacts positively on accuracy of templating in hip arthroplasty.

Evidence in the literature suggests that templating accuracy may be further improved with the use of more modern ECDs. A systematic review by Alnahhal et al. (2019) found that the KingMark device had higher rates of templating accuracy than the single marker ball [1]. Similarly, Al-Ashqar et al. (2021) conducted a retrospective analysis of templating accuracy, specifically finding improved levels of accuracy of cup size using the KingMark [18]. Interestingly, this finding is contradicted by Maatough et al. (2025), who found that the KingMark showed improved accuracy in templating stem sizes but specifically not acetabular cups [5]. Similarly, Girgis et al. (2023), during their assessment of accuracy of digital templating methods, found that the addition of the KingMark device made only a slight difference in accuracy overall, with KingMark actually leading to marginally lower exact femoral component size accuracy compared to templating with no radiographic marker (65.3% vs. 67.2%, respectively) [19]. Another described technique is the DSCM method, where one reflective marker is moved through space to define two different scale factors, allowing the calculation of two separate calibration scales. In an observational study, Ries et al. (2023) found improved magnification factor differences with DSCM as opposed to a single marker ball, and additionally suggest that patients find the DSCM method physically more comfortable [20]. Loweg et al. (2020) also found the DSCM technique had superior results over other ECDs, producing a more accurate calibration factor [3]. It may be worthwhile for centres using single marker ball ECDs to consider utilising a more up-to-date device.

It was noted that audit standard compliance was not to the desired level in the first cycle, and that it deteriorated further in the second cycle. Therefore, whilst physical marker ball placement improved overall, there was an increase in the number of pre-operative pelvic X-rays with an absent or partially visible marker ball and therefore a reduction in compliance. Possible reasons for this include variability between radiology technicians [5], difficulty positioning a patient in pain or with cognitive impairment [3] or a clinical history not suggestive of trauma, meaning the marker ball was not included. It is important that each hospital radiology department ensures that every initial pelvic X-ray in the context of a fall, trauma, or hip pain contains the preferred external calibration marker as per the local trauma and orthopaedic team.

There are multiple possible limitations of this closed-loop audit. Educational measures led to a statistical improvement in marker ball placement, and therefore templating accuracy, but we are yet to re-assess with a third audit cycle. It may be that correct physical marker ball placement has been subject to attrition over time, and further teaching sessions may be necessary to maintain good practice. Our sample sizes in each cycle were relatively small and solely from one centre, which could affect statistical power; we are a district general hospital, so operative numbers are generally smaller than in larger centres. Additionally, a significant number of hip arthroplasties initially identified in each cycle were excluded as pre-operative radiographs were not templated by the operating clinician; in the second cycle, 19% of THAs and hemiarthroplasties for trauma were not templated, automatically excluding them from analysis. This could possibly have produced a limitation bias. Personal clinical practice varies, but ideally there should be a general consensus within a department on whether templating with ECDs is performed. Another limitation is that we chose to focus only on femoral head size to simplify the project, so the data do not assess whether correct marker ball placement impacts upon accuracy of femoral stem or acetabular cup size. There are also other factors that may affect marker ball positioning that we were unable to adjust for, such as patient factors (pain at the time of X-ray, confusion), radiographer experience, and patient body habitus.

Overall, further audit cycles would be required to ensure standards are upheld in our Trust with ongoing radiology education, and further research with larger sample sizes would be required to fully elucidate the clinical impact that improved accuracy of templating may bring, including potential effects on reduction of complications, surgical efficiency, and the stocking of certain prosthesis sizes. This could then ensure that our results were fully generalisable to clinical practice rather than specifically to our Trust.

## Conclusions

This quality improvement project has demonstrated that simple educational measures can positively impact radiological marker ball placement, in this case leading to a significant improvement in accuracy of templating head size in hip arthroplasty surgery. Despite an increase in accuracy of the templated head size, in the second cycle, the compliance of correctly positioned marker balls in the radiographs did decrease. This would be an area to address through further departmental education in the future. Regular re-evaluation in the form of audit and frequent teaching is crucial to maintaining high standards of clinical practice. In centres that choose to utilise them, correct placement of the marker ball is key to accurate pre-operative templating and prosthesis implantation, highlighting the importance of multi-disciplinary collaboration in effective and safe patient care.

## Figures and Tables

**Figure 1:**
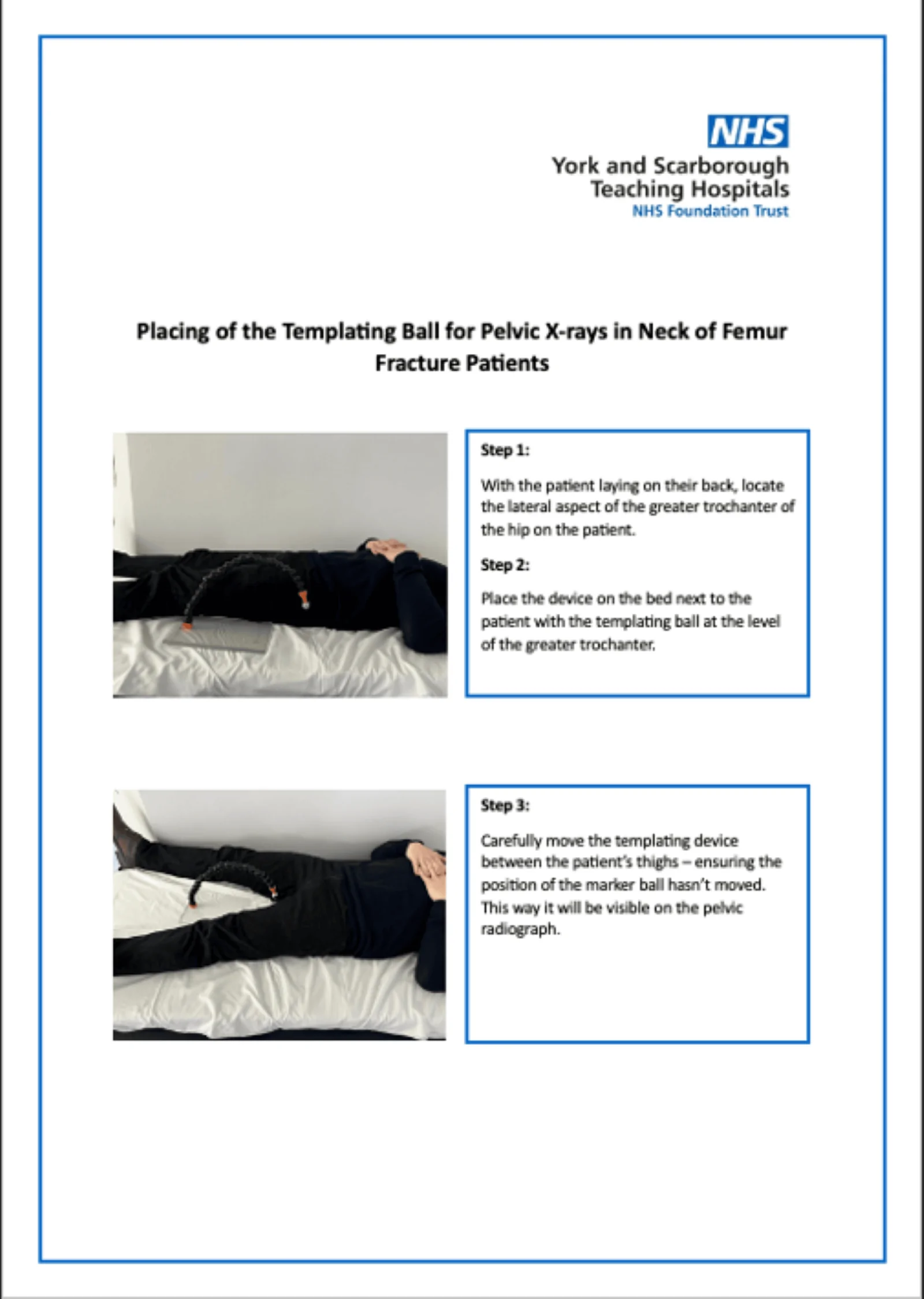
Poster distributed in the Radiology Department.
